# Optimal usage of the GnRH antagonists: a review of the literature

**DOI:** 10.1186/1477-7827-11-20

**Published:** 2013-03-15

**Authors:** Alan B Copperman, Claudio Benadiva

**Affiliations:** 1 Mount Sinai Medical Center, New York, NY, USA; 2 Reproductive Medicine Associates of New York, New York, NY, USA; 3 The Center for Advanced Reproductive Services, Department of Ob/Gyn, University of Connecticut, Farmington, CT, USA

**Keywords:** GnRH antagonists, GnRH agonists, IVF, Ovarian stimulation, OHSS

## Abstract

Gonadotropin-releasing hormone (GnRH) antagonists, which became commercially available from 1999, have been used for the prevention of premature luteinizing hormone (LH) surges in controlled ovarian stimulation for in vitro fertilization or intracytoplasmic sperm injection. This review focuses on the recent literature on the use of GnRH antagonists and provides guidelines for optimal use in light of increasing evidence showing that GnRH antagonists are safe and effective, allowing flexibility of treatment in a wide range of patient populations. This includes patients undergoing first-line controlled ovarian stimulation, poor responders, and women diagnosed with polycystic ovary syndrome. The GnRH antagonist offers a viable alternative to the long agonists, providing a shorter duration of treatment with fewer injections and with no adverse effects on assisted reproductive technology outcome. This results in a significantly lower amount of gonadotropins required, which is likely to lead to improved patient compliance.

## Background

Gonadotropins were first introduced in the early 1960s and have been used in ovarian stimulation cycles to induce multiple follicular development, particularly during the past 3 decades, in women undergoing in vitro fertilization (IVF) treatment. Gonadotropin-releasing hormone (GnRH) analogs are administered along with gonadotropins to prevent the occurrence of a surge in luteinizing hormone (LH), which may occur prematurely before the leading follicle reaches the optimum diameter (≥17 mm) for triggering ovulation by human chorionic gonadotropin (hCG) injection. Without the use of GnRH analogs, LH surges would occur in approximately 20% of stimulated IVF patients [1,2]. Preventing LH surges using GnRH analogs improves oocyte yield with more embryos, allowing better selection and, therefore, leading to an increase in pregnancy rates [3].

Since the early 1980s, the use of GnRH agonists in ovarian stimulation has greatly improved the success rate of IVF [4]. GnRH agonists reduce the incidence of premature LH surges [5,6] by suppressing gonadotropin release via pituitary desensitization following an initial short period of gonadotropin hypersecretion. More recently, GnRH antagonists with high potency and fewer side effects have been introduced into IVF and have emerged as an alternative in preventing premature LH surges. Unlike GnRH agonists, these potent GnRH antagonists cause immediate, rapid gonadotropin suppression by competitively blocking GnRH receptors in the anterior pituitary gland, thereby preventing endogenous GnRH from inducing LH and follicle-stimulating hormone (FSH) release from the pituitary cells. Furthermore, GnRH antagonist suppression of gonadotropin secretion can be quickly reversed [7-9]. This different pharmacologic mechanism of action makes GnRH antagonists a more logical choice to use in IVF for the prevention of premature LH surges [5].

**Figure 1:**
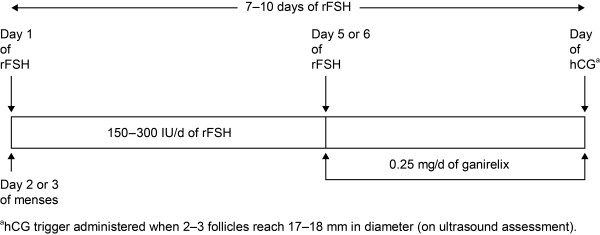
Schematic presentation of the ganirelix treatment regimen.

Ganirelix (Orgalutran, N.V. Organon, Oss, The Netherlands) and cetrorelix (Cetrotide, Serono International S.A., Geneva, Switzerland) are subcutaneously administered GnRH antagonists approved for clinical use in IVF therapy [10,11]. Since 1999, GnRH antagonists have been used for the prevention of premature LH surges in women undergoing controlled ovarian stimulation for IVF. Clinically, stimulation with urinary FSH or recombinant human FSH (rFSH), either alone or in combination with urinary-derived human menopausal gonadotropin (hMG), is started on day 2 or 3 of the menstrual cycle and the GnRH antagonist is administered in the late follicular phase, from day 5 or 6 of stimulation onward. The dose of gonadotropins may be adjusted according to individual response. Both gonadotropins are continued daily until two to three follicles reach ≥17 mm in diameter (on ultrasound assessment) at which time hCG is administered to induce final oocyte maturation (Figure 1). This review focuses on literature concerning the use of GnRH antagonists in ovarian stimulation for IVF and provides guidelines for optimal use.

### Potential advantages of GnRH antagonist protocols

There are a number of theoretical advantages of GnRH antagonists versus GnRH agonists [12,13], including a shorter duration of injectable drug treatment, absence of vasomotor symptoms, less risk of inadvertent administration during early pregnancy, avoidance of ovarian cyst formation, and a significantly smaller dose of gonadotropin per cycle, which translate to improved patient convenience [9,14]. The literature regarding the cost effectiveness of GnRH antagonist protocols is currently contradictory. In a randomized trial by Badrawi et al. [15], the cost of medication per cycle and per pregnancy was shown to be higher in a GnRH antagonist protocol than a GnRH agonist protocol, while an observational study by Kamath et al. [16] found costs of the two protocols to be similar.

Current evidence suggests that GnRH antagonists and agonists are similarly effective in the context of oocyte donation [17]. However, due to their increased convenience, GnRH antagonist protocols are often the regimen of choice for oocyte donors. More recently, it has been recommended that treatment guidelines for the prevention of ovarian hyperstimulation syndrome (OHSS) [18] should be updated to incorporate findings from the literature over the past 5 years. The literature shows that GnRH antagonist protocols and GnRH agonist triggering of final oocyte maturation, especially when used in combination, may reduce OHSS and have considerable promise in preventing OHSS [19].

### Potential disadvantages of GnRH antagonist protocols

Potential disadvantages of GnRH antagonist protocols over GnRH agonist protocols include less flexible options in terms of cycle programming and early studies suggesting a minor reduction in pregnancy rates per cycle [20,21]. Increasing flexibility of GnRH antagonist protocols can be achieved with oral contraceptives [20]. Pretreatment with oral contraceptives allows programming of cycles, whereby stimulation can be started during a 5-day interval following withdrawal of the oral contraceptive [22]. Use of oral contraceptives with a GnRH antagonist protocol and the pregnancy outcomes of GnRH antagonist protocols are discussed below.

### Pregnancy outcomes of GnRH antagonist protocols

Despite an initial trend toward a lower pregnancy rate with GnRH antagonists compared with agonists in a number of early randomized controlled studies, a meta-analysis by Kolibianakis et al. [23] and a review by Tur-Kaspa and Ezcurra [24] found no significant difference in the probability of live birth rates with the use of either a GnRH agonist or antagonist protocol [23] (Table 1).

**Table 1:** Results of meta-analyses of GnRH analogs among patients treated for IVF – odds ratio of live birth rate

	***GnRH antagonists***		***GnRH agonists***		***Weight***	***Odds ratio (95% CI)***
	***Events***	***Total***	***Events***	***Total***		
RCTs included in Kolibianakis et al. [23]						
Albano 2001 [25]	34	198	19	95		0.83 (0.44-1.55)
European 2000 [8]	97	486	61	244		0.75 (0.52–1.08)
Olivennes 2000 [26]	22	126	9	43		0.80 (0.34–1.90)
N American 2001 [27]	60	208	36	105		0.78 (0.47–1.28)
Middle East 2001 [28]	72	236	37	119		0.97 (0.60–1.57)
Akman 2001 [29]	4	24	5	24		0.76 (0.18–3.26)
Hohmann 2003 [30]	18	111	10	58		0.93 (0.40–2.17)
Martinez 2003 [31]	4	21	3	23		1.57 (0.31–8.01)
Franco 2003 [32]	3	14	2	6		0.55 (0.07–4.56)
Hwang 2004 [33]	8	27	8	29		1.11 (0.35–3.53)
Sauer 2004 [34]	9	24	9	25		1.07 (0.33–3.41)
Loutradis 2004 [35]	9	58	12	58		0.70 (0.27–1.63)
Check 2004 [36]	8	30	5	30		1.82 (0.52–6.38)
Xavier 2005 [37]	7	66	8	65		0.85 (0.29–2.48)
Malmusi 2005 [38]	5	30	5	30		1.00 (0.28–3.89)
Marci 2005 [39]	4	30	0	30		10.38 (0.53–201.45)
Cheung 2005 [40]	3	33	2	33		1.55 (0.24–9.94)
Barmat 2005 [41]	13	40	17	40		0.65 (0.26–1.62)
Bahceci 2005 [42]	29	73	33	75		0.84 (0.44–1.61)
Badrawi 2005 [15]	11	50	13	50		0.80 (0.32–2.02)
Schmidt 2005 [43]	3	24	3	24		1.00 (0.18–5.53)
Lee 2005 [44]	13	41	8	20		0.70 (0.23–2.11)
**Total (n = 22)**	**436**	**1950**	**305**	**1226**		**0.86 (0**.**72–1**.**02)**
RCTs included in Al-Inany et al. [45]						
**All women**						
Albano 2000 [25]	34	198	19	95	13.5%	0.83 (0.44–1.55)
Barmat 2005 [41]	13	40	17	40	7.3%	0.65 (0.26–1.62)
Heijnen 2007 [46]	70	205	78	199	33.0%	0.80 (0.54–1.21)
Hurine 2006 [47]	17	91	17	91	8.8%	1.00 (0.47–2.11)
Kim 2009 [48]	13	54	8	28	5.1%	0.79 (0.28–2.22)
Kurzawa 2008 [49]	14	37	18	37	7.1%	0.64 (0.25–1.62)
Lin 2006 [50]	22	60	21	60	8.4%	1.08 (0.51–2.27)
Marci 2005 [39]	4	30	0	30	0.3%	10.36 (0.53–201.45)
Ye 2009 [51]	35	109	39	111	16.6%	0.87 (0.50–1.53)
**Subtotal (95% CI)**		**824**		**691**	**100.0%**	**0.86 (0**.**69–1.08)**
Total events	222		217			
Heijnen 2007 [46]	70	205	78	199	79.7%	0.80 (0.54–1.21)
Lin 2006 [50]	22	60	21	60	8.4%	1.08 (0.51–2.27)
**Subtotal (95% CI)**		**265**		**259**	**100.0%**	**0.89 (0**.**62–1.26)**
Total events	97		102			
Heterogeneity: χ^2^ = 0.32, *df* = 1 (P = 0.57)						
Test for overall effect: *Z* = 0.66 (P = 0.51)						
**Cetrorelix only**						
Albano 2000 [25]	34	198	19	95	26.3%	0.83 (0.44–1.55)
Hurine 2006 [47]	17	91	17	91	17.1%	1.00 (0.47–2.11)
Kim 2009 [48]	13	54	8	28	9.9%	0.79 (0.28–2.22)
Kurzawa 2008 [49]	14	37	18	37	13.8%	0.64 (0.25–1.62)
Marci 2005 [39]	4	30	0	30	0.5%	10.36 (0.53–201.45)
Ye 2009 [51]	35	109	39	111	32.4%	0.87 (0.50–1.53)
**Subtotal (95% CI)**		**519**		**392**	**100.0%**	**0**.**89 (0.65–1.23)**
Total events	97		102			
Heterogeneity: χ^2^ = 3.31, *df* = 5 (P = 0.65)						
Test for overall effect: *Z* = 0.70 (P = 0.49)						
**Ganirelix only**						
Barmat 2005 [41]	13	40	17	40	100.0%	0.65 (0.26–1.62)
**Subtotal (95% CI)**		**40**		**40**	**100.0%**	**0.65 (0.26–1.62)**
Total events	97		102			
Heterogeneity: not applicable						
Test for overall effect: *Z* = 0.70 (P = 0.36)						

Adapted from Kolibianakis 2006 [23] and Al-Inany 2011 [45].

GnRH = gonadotropin-releasing hormone; IVF = in vitro fertilization.

In normal responders, the use of GnRH antagonist versus long GnRH agonist protocols was associated with a statistically significant reduction of OHSS, with no evidence of a difference in live birth rates [45]. GnRH antagonist protocols have been shown to result in better outcomes than GnRH agonists in patients with poor prognosis [52,53]. In a meta-analysis of six clinical trials comparing GnRH antagonist versus GnRH agonist protocols in poor ovarian responders in IVF/intracytoplasmic sperm injection (ICSI) cycles Franco et al. [54] indicated no difference between GnRH antagonists and agonists with respect to cycle cancellation rate, number of mature oocytes, and clinical pregnancy rate per cycle initiated, per oocyte retrieval, and per embryo transfer. Al-Inany et al. [45] found no significant difference following the use of GnRH antagonist and agonist protocols in a recent Cochrane review.

In oocyte donation [55] and embryo transfer [56] cycles, the replacement of GnRH agonist with a GnRH antagonist had no impact on the pregnancy and implantation rates. Higher pregnancy rates were also shown in a gonadotropin intrauterine insemination cycle than in a cycle where no intervention took place [57]. In a prospective randomized trial, Prapas et al. [58] reported that GnRH antagonist administration during the proliferative phase did not adversely affect endometrial receptivity in oocyte recipients.

### Optimal use of GnRH antagonists in diverse treatment situations

#### First-line treatment

GnRH antagonists have been shown to be an effective treatment in women undergoing controlled ovarian stimulation for IVF in multiple meta-analyses and clinical studies. In the systematic review and meta-analyses by Kolibianakis et al. [23], it was shown that the probability of live birth was not dependent on the type of GnRH analog used for the suppression of premature LH rises (odds ratio 0.86; 95% confidence interval 0.72-1.02). In a more recent systematic review, Al-Inany et al. [45] also reported that there was no significant difference in live birth rates following a GnRH antagonist or GnRH agonist protocol (odds ratio 0.86, 95% confidence interval 0.69-1.08).

In a retrospective review of patients with good prognosis undergoing their first IVF cycle, Johnston-MacAnanny et al. [59] showed that clinical and ongoing pregnancy rates and implantation rates were similar in 755 good responder patients undergoing a GnRH agonist protocol and 378 good responder patients undergoing a GnRH antagonist protocol during their first cycle of IVF. Borm and Mannaerts [8] evaluated the efficacy and safety of ganirelix in 730 women undergoing ovarian stimulation with rFSH. The patients were randomized in a 2:1 ratio to either 0.25 mg ganirelix or buserelin (the trial was designed as a noninferiority study using a long protocol of intranasal buserelin and rFSH as a reference treatment). Ganirelix in comparison with buserelin resulted in a shorter duration of treatment (5 vs 26 days). Comparison of the number and size of follicles indicated that in the ganirelix group, the final number of follicles on the day of hCG administration, was smaller (10.7 vs 11.8) and produced less peak estradiol concentration (1190 vs 1700 pg/ml) than the buserelin group. The ganirelix regimen resulted in the recovery of good-quality oocytes, as reflected by the high fertilization rate (62.1%), and a similar number of good-quality embryos (3.3), as the reference group (3.5). The clinical outcome (defined as the ongoing pregnancy rate per attempt) was good (20.3%), although pregnancy rates were found to be slightly higher in the reference group (25.7%). Interestingly, the ongoing pregnancy rate per attempt for patients treated at study sites (n = 10) that had previous experience with the ganirelix regimen was similar, that is, 24.2% in the ganirelix group vs 23.6% in the buserelin group. This suggests that the slightly lower pregnancy rates observed in early trials may have been related to lack of experience with the use of antagonist protocols. With regard to safety, ganirelix was found to be safe and well tolerated with a two-fold lower (2.4%) incidence of OHSS than was found in the buserelin (5.9%) group. Overall, the study demonstrated that ganirelix provides a safe, short, and convenient treatment option for patients undergoing controlled ovarian hyperstimulation for IVF/ICSI and results in good clinical outcome.

#### Second-line treatment (treatment of poor responders)

GnRH antagonists have been used effectively in patients who have a poor prognosis or who have shown a diminished ovarian response to controlled ovarian stimulation. In the systematic review and meta-analyses by Kolibianakis et al. [23], it was shown that the probability of live birth in poor responders was not dependent on the type of GnRH analog used for the suppression of premature LH rises (odds ratio 1.34; 95% confidence interval 0.70-2.59). In a more recent systematic review, Al-Inany et al. [45] also reported no significant differences in clinical pregnancy rates in poor responders following a GnRH antagonist and GnRH agonist protocol (odds ratio 0.71, 95% confidence interval 0.49-1.02).

Schmidt et al. [43] showed that the use of GnRH antagonists was as effective as the conventional microdose protocol and that embryo quality, implantation rates, and ongoing pregnancy rates were comparable in a randomized prospective study comparing ganirelix with a microdose GnRH agonist in patients with poor ovarian response. The microdose flare protocol has been proven to increase both clinical and ongoing pregnancy rates in poor responders. The authors concluded that the ganirelix protocol may be preferable because it requires significantly fewer injections and a shorter treatment course, resulting in cost savings and improved convenience for the patient. An earlier review by Copperman [60] also noted that the use of a GnRH antagonist for the suppression of premature LH surges in poor responders is at least as good as the microdose flare and provides better cycle outcomes than the long luteal leuprolide acetate downregulation protocols.

The use of GnRH antagonists among patients with poor prognosis was also evaluated by Shapiro et al. [12] in a nonrandomized, noncontrolled, retrospective review of 204 patients (165 cycles in patients with a normal IVF prognosis and 60 cycles in those with a poor prognosis). Overall, the pregnancy rates per initiated cycle and per embryo transfer were 33.3% and 42.1%, respectively, with a cycle cancellation rate of 21%. The patients with poor prognosis had a pregnancy rate of 8.3% per attempt and 15% per transfer compared with 40% and 45%, respectively, in patients with normal prognosis. While this retrospective analysis supports the use of GnRH antagonist protocols as an alternative to agonist protocols in normal responders, the use of GnRH antagonists in patients with poor IVF prognosis resulted in predictably poor outcomes.

In a recent meta-analysis comparing the efficacy of GnRH antagonists versus agonists in poor responders, Pu et al. [61] showed that GnRH antagonists resulted in a shorter duration of stimulation, but there was no difference in the number of oocytes retrieved, the cycle cancellation rate, or the clinical pregnancy rate.

The ability to offer patients who have suffered numerous failed cycle attempts a choice of effective alternatives may improve outcomes for these women. Currently, in many centers, the luteal phase estradiol patch/GnRH antagonist (LPG) protocol is the treatment of choice for women with a poor response to ovarian stimulation. This protocol involves administration of transdermal estradiol patches and a GnRH antagonist in the luteal phase of the preceding menstrual cycle, followed by high-dose follicular phase gonadotropin stimulation with adjunctive GnRH antagonist. Dragisic et al. [62] first described this novel protocol in 2005 and demonstrated that it improved ovarian responsiveness among poor responders, with more uniform follicular development, more oocytes retrieved, higher number of transferred embryos, and improved pregnancy rates. Weitzman et al. [63] retrospectively compared the outcomes of patients with a history of failed cycles who had undergone ovarian stimulation with either an LPG protocol (n = 45) or a microdose agonist protocol (n = 76) over a 1-year period [63]. All clinical outcomes, including ongoing pregnancy rates, were comparable between the two groups, suggesting that the use of an LPG protocol is at least as effective as a microdose agonist protocol. Similar findings were obtained by the same group of investigators in a subsequent prospective randomized controlled trial (RCT) [64].

### Dosing schedules

#### Single dose

Cetrorelix acetate, a US Food and Drug Administration-approved GnRH antagonist, has been shown to be effective and safe as a single-dose (3 mg) or multiple-dose regimen (0.25 mg daily) [65,66]. In a prospective randomized trial, Vlaisvljevic et al. [67] showed that 3 mg cetrorelix had comparable efficacy to the GnRH agonist goserelin. However, multiple-dose protocols are now the standard and single-dose protocols are rarely used.

#### Multiple dose

Ganirelix is only available as a multiple-dose regimen. The multiple-dose protocol for ganirelix involves the administration of 0.25 mg daily from day 6 or 7 of stimulation, or when the leading follicle is 14–15 mm, until hCG administration [68]. The Ganirelix Dose-Finding Study [69] was the first multicenter, double-blind, randomized dose-finding study to establish the minimal effective dose of ganirelix to prevent premature LH surges in 333 women undergoing ovarian stimulation with rFSH. Six different ganirelix doses (0.0652, 0.125, 0.25, 0.5, 1.0, and 2.0 mg/0.5 ml) were administered daily by subcutaneous injection. In this study, patients were treated with a fixed dose of 150 IU rFSH for 5 days before starting ganirelix. The study revealed that 0.25 mg/d was the minimal effective dose with regard to preventing LH surges and resulted in a good clinical outcome with an ongoing pregnancy rate of 34% per attempt and 37% per transfer. Administration of 0.25 mg daily ganirelix has been shown to be safe and effective in the prevention of premature LH surge in further studies [8,27,28].

The North American Ganirelix Study Group administered this GnRH antagonist to 313 patients for whom controlled ovarian hyperstimulation and IVF/ICSI were indicated [27]. Patients received one controlled ovarian hyperstimulation cycle with ganirelix or a long protocol of leuprolide acetate in conjunction with rFSH [27]. From day 6 of rFSH treatment, ganirelix (0.25 mg) was administered daily up to and including the day of hCG administration. The mean number of oocytes retrieved per attempt was 11.6 in the ganirelix group and 14.1 in the leuprolide group. Fertilization rates were 62.4% and 61.9% and implantation rates were 21.1% and 26.1% in the ganirelix and leuprolide groups, respectively. Clinical and ongoing pregnancy rates per attempt, respectively, were 35.4% and 30.8% in the ganirelix group and 38.4% and 36.4% in the leuprolide acetate group. Fewer moderate and severe injection-site reactions were reported with ganirelix (11.9% and 0.6%) than with leuprolide (24.4% and 1.1%).

The European and Middle East Orgalutran Study Group, compared the use of ganirelix (0.25 mg administered from day 6 of rFSH treatment up to and including the day of hCG administration) with the GnRH agonist triptorelin (0.1 mg), as a reference treatment in a long protocol [28]. Overall, the results showed that ganirelix achieved similar clinical efficacy with a shorter duration of treatment compared with the GnRH agonist. The ganirelix regimen was 17 days shorter (9 vs 26 days) than the triptorelin regimen with a median reduction in the total dose of rFSH utilized of 450 IU (1350 vs 1800 IU). The fertilization rates and the number of good-quality embryos were similar in both treatment groups. The implantation rates of the two treatment arms were identical (22.9%) with similar ongoing pregnancy rates (31.0% for ganirelix vs 33.9% for triptorelin). Furthermore, local tolerance of ganirelix appeared to be better than that of triptorelin, as the percentage of subjects with at least one local skin reaction was two-fold lower when using the ganirelix regimen (11.9% for ganirelix vs 24.1% for triptorelin).

In a prospective randomized trial in 185 patients undergoing assisted reproductive technologies Wilcox et al. [70] compared a single injection of cetrorelix (3 mg) with a daily dose of 0.25 mg of ganirelix in a flexible protocol. Cetrorelix and ganirelix were found to be equally effective; no patient in either treatment group had a premature LH surge and there were no statistically significant differences between treatments for any IVF/ICSI outcomes, including pregnancy rates. Cetrorelix is also available as a multiple-dose regimen (0.25 mg daily). Hsieh et al. [71] reported that the minimum effective dose of cetrorelix for pituitary suppression is 0.25 mg, resulting in comparable pregnancy rates. Olivennes et al. [65] showed that the multiple-dose regimen of cetrorelix (0.25 mg daily) offers equal efficacy and safety to the single-dose regimen (3 mg). Similar efficacy and safety results were shown in a cetrorelix (0.25 mg daily) or buserelin protocol [21].

#### Flexible versus fixed dosing

Flexible dosing was introduced to reduce the number of antagonist injections and the duration of stimulation. It is recommended that fixed dosing is started from day 5 or 6 of stimulation [72,73] while flexible dosing starts when the follicles reach a size of >14 mm [74-76]. It has been suggested that development of flexible dosing regimens, that is, individualizing or tailoring GnRH antagonist administration, might lead to better clinical outcomes in GnRH antagonist-treated patients [77]. Results from several clinical studies support the efficacy and safety of flexible-dosing regimens with ganirelix, though some show no significant advantage over the standard fixed-dose regimen [78-80].

Nevertheless, there is evidence that flexible dosing regimens lead to improvement in the outcomes of ovarian stimulation cycles. In a prospective, randomized, single-center study comparing fixed multiple-dose antagonist with a flexible ganirelix regimen, Ludwig et al. [75] showed an improved outcome when a tailored rather than a standardized fixed protocol was used to schedule the start of the GnRH antagonist; a higher yield of oocytes was achieved despite less rFSH used. There were, however, no differences in pregnancy rates among the three groups.

The benefits of flexible GnRH antagonist administration according to follicular size versus starting dosing on a fixed day were also highlighted by Al-Inany et al. [81] in a meta-analysis of four randomized trials. Although no statistically significant difference in pregnancy rate was observed between flexible and fixed protocols, there was a significant reduction in the amount of rFSH with the flexible protocol.

### Use with GnRH agonist trigger

Ovulation can either be induced with a bolus injection of hCG or a GnRH agonist. An advantage of using a GnRH agonist to trigger final oocyte maturation is the potential reduction in the risk of OHSS [22]. As an effective alternative to hCG-induced ovulation, GnRH agonists induce a sustained release of LH (and FSH) from the pituitary that effectively induces oocyte maturation and ovulation. A possible advantage of a GnRH agonist for trigger in comparison with hCG is the simultaneous induction of a FSH surge comparable to the surge of a natural cycle [82]. GnRH agonist triggering, however, results in a shorter endogenous LH surge that leads to a defective corpus luteum formation and an inadequate luteal phase [83,84]. The profound luteolysis observed after GnRH agonist triggering in contrast to the prolonged luteotropic effect often seen after triggering with hCG has been shown to almost completely eliminate the risk of OHSS in high responders, avoiding the need for cycle cancellation [82,85]. Because of the inadequate luteal phase after GnRH agonist trigger, the use of standard luteal phase support is inadequate and results in lower conception and higher miscarriage rates [86]. Therefore, luteal support strategies including one bolus of low-dose hCG, repeated boluses of hCG, recombinant LH add-back, and more intensive estradiol and progesterone supplementation were proposed to achieve optimal conception rates [82,87-89].

Engmann et al. [87] showed that this approach was effective in a clinical study in which 66 patients at high risk for developing OHSS were randomized to an ovarian stimulation protocol consisting of either a GnRH agonist trigger after co-treatment with ganirelix or a control group that received hCG trigger after dual pituitary suppression with birth control pills and a GnRH agonist. None of the patients receiving ganirelix developed OHSS compared with 31% of the patients in the control group. The study also found no significant differences in the rates of implantation (36.0% with ganirelix vs 31.0% with control), clinical pregnancy (56.7% vs 51.7%), and ongoing pregnancy (53.3% vs 48.3%). In concluding, the authors suggested that a protocol consisting of a GnRH agonist trigger after GnRH antagonist co-treatment combined with luteal phase and early pregnancy estradiol and progesterone supplementation should be given strong consideration for patients at high risk of developing OHSS.

In a more recent publication reviewing the predictive factors of successful outcome after GnRH agonist trigger and intensive luteal support, Kummer et al. [90] identified serum LH on the day of trigger and peak estradiol levels ≥4000 pg/ml as the most important predictors of success. Women with peak estradiol ≥4000 pg/ml had a significantly higher clinical pregnancy rate than women with peak estradiol <4000 pg/ml after GnRH agonist trigger (53.6% vs 38.1%) [90]. The same group of investigators subsequently reported that a dual trigger of final oocyte maturation with a GnRH agonist and low-dose hCG (1000 IU) resulted in improved implantation, clinical pregnancy, and live birth rates compared with a GnRH agonist alone, without increasing the risk of clinically significant OHSS in patients with peak estradiol levels <4000 pg/ml [91].

Griesinger et al. [92] investigated the effect of cryopreservation of all two pronuclei-stage zygotes following GnRH agonist trigger on the incidence of severe OHSS and ongoing pregnancy rate in a prospective, observational, proof-of-concept study. The ongoing pregnancy rate was 36.8% (95% confidence interval 19.1–59.0). No patients developed moderate or severe OHSS [92].

### LH add-back

Despite the advantages of GnRH antagonists—that is, much shorter treatment regimens, fewer injections, and the need for less gonadotropin—the more general acceptance of antagonist regimens has been hampered by their perceived association with slightly lower pregnancy and implantation rates compared with GnRH agonist protocols.

Results from early studies suggested that low implantation rates were due to high daily doses of GnRH antagonists (0.5, 1, or 2 mg once daily) inducing a sharp decrease in serum LH concentrations during the follicular phase of ovarian stimulation [93,94]. Supplementation with exogenous recombinant human LH (rLH) was suggested as an alternative to counter the consequences of LH depletion. In an RCT that included 60 patients, Garcia-Velasco et al. [95] employed an innovative protocol in which the pituitary response was suppressed with high-dose GnRH antagonist and rLH was added back to correct the diminished implantation rate. GnRH antagonist treatment (2 mg/d) was initiated on day 6 of stimulation together with 375 IU rLH, and maintained until the day of hCG administration, while control subjects received the standard dose of 0.25 mg/d. Fluctuating LH concentrations were present on days 3 and 6 in both groups. This fluctuation continued on day 8 and on the day of hCG administration in the control (low-dose) group, where 30% of patients had LH concentrations <1 IU/L on the hCG day. The study (high-dose) group showed stable LH concentrations on day 8 and on the hCG day, with no LH surges. No clinical differences in outcomes were found between the treatment groups. The LH add-back strategy (375 IU/d) appeared to “rescue” the adverse effects that high doses of GnRH antagonist have been seen to impose on implantation.

More recently, Bosch et al. [96] assessed the impact of LH add-back on cycle outcome during ovarian stimulation with GnRH antagonists in an RCT performed in two age subgroups ≤35 years (n = 380) and 36–39 years (n = 340). rLH administration significantly increased the implantation rate in the older population, and a clinically relevant (although not statistically significant) better ongoing pregnancy rate per cycle was observed. Interestingly, no benefit from rLH administration was demonstrated in patients younger than 36 years.

### Use with and without oral contraceptives

Oral contraceptive pill pretreatment in GnRH antagonist cycles has been advocated for scheduling ovarian stimulation and oocyte retrieval in IVF programs. An RCT, by Rombauts et al. [97], assessed the impact of oral contraceptive scheduling with a ganirelix regimen on the ovarian response of women undergoing rFSH stimulation for IVF, compared with a nonscheduled ganirelix regimen and a long GnRH agonist (nafarelin) protocol. The study found that in the three groups the number of oocytes retrieved and the number of good-quality embryos were similar. Evidence from several other RCTs in the literature supports the use of oral contraceptive scheduling and shows that success rates are the same [98,99], although it has been found that after oral contraceptive pretreatment it may take an extra day to stimulate [100]. In the most recent Cochrane review, a subgroup analysis of 10 RCTs that used oral contraceptives pretreatment showed that there were no significant differences in ongoing pregnancy rates in GnRH antagonist protocols compared with GnRH agonist protocols [45].

Conversely, Griesinger et al. [101] showed a statistically significant reduction in the likelihood of ongoing pregnancy with oral contraceptive pretreatment when a pill-free interval of 2–5 days is used before starting gonadotropin stimulation in a meta-analysis of six RCTs on oral contraceptive pretreatment in GnRH antagonist IVF cycles involving 1343 patients. The negative effect of the oral contraceptive pretreatment on the IVF outcome may be explained by the fact that some of the studies included in the meta-analysis [101] started ovarian stimulation 2–3 days after the last oral contraceptive pill rather than 5 days later.

More research is needed to determine the most reliable and efficacious way to schedule GnRH antagonist stimulation cycles with oral contraceptive pretreatment.

### Use with and without estrogen pretreatment

Estrogen pretreatment in GnRH antagonist cycles has also been suggested as an alternative method to achieve gonadotropin suppression during the early follicular phase so that scheduling ovarian stimulation and oocyte retrieval in IVF programs can be planned. Guivarc’h-Levêque et al. [102] found that estrogen pretreatment was safe in a large prospective study of patients undergoing IVF/ICSI. A greater requirement of FSH and a longer duration of stimulation were associated with estrogen pretreatment [103,104]. However estrogen pretreatment did not affect the IVF/ICSI outcomes [103].

### Decreasing OHSS with GnRH antagonists

OHSS is a preventable condition and implementing evidence-based prevention strategies should enable clinicians to reduce its occurrence. As we have discussed, GnRH antagonist protocols and the use of a GnRH agonist to trigger final oocyte maturation in a GnRH antagonist protocol are two treatment strategies that could reduce or prevent OHSS, especially when used in conjunction.

Significantly elevated or rapidly rising serum estradiol concentrations are known to predispose patients to development of OHSS. Therefore, since GnRH antagonist treatment is associated with reduced estradiol concentrations, it might be expected to decrease the risk of OHSS [105]. Gustofson et al. [105] showed that ganirelix treatment rapidly reduced circulating estradiol concentrations without adversely affecting oocyte maturation, fertilization rates, or embryo quality and resulted in a high pregnancy rate in the subgroup of women with ovarian hyper-response who were pretreated with a GnRH agonist. Despite the treatment cohort being at high risk of developing OHSS, only two cases of severe OHSS occurred. The RCT by Lainas et al. [106] provided further evidence to support the use of GnRH antagonists among patients at risk of OHSS. This study compared a flexible GnRH antagonist protocol with the long GnRH agonist down-regulation protocol in 220 patients with polycystic ovary syndrome (PCOS). While pregnancy rates were similar in the two protocols, the GnRH antagonist protocol was associated with a significantly lower incidence of OHSS.

The reduction in the incidence of OHSS with GnRH antagonist protocols was shown in the Cochrane review of 27 RCTs in 2006 [107] and 29 RCTs in 2011 [45]. These systematic reviews compared GnRH antagonists (ganirelix or cetrorelix) with the long protocol of GnRH agonist. A statistically significant reduction in the incidence of severe OHSS with the antagonist protocol (27 RCTs: relative risk ratio, 0.61; 95% confidence interval, 0.42–0.89; *P* = 0.01 [107]; 29 RCTs: odds ratio 0.43, 95% confidence interval 0.33–0.57 [45]) was observed. In addition, interventions to prevent OHSS, such as coasting and cycle cancellation, were administered more frequently in the agonist group (27 RCTs: odds ratio, 0.44; 95% confidence interval, 0.21–0.93; *P* = 0.03 [107]; 29 RCTs: odds ratio, 0.50, 95% confidence interval, 0.33–0.76; *P* = 0.001 [45]). In a meta-analysis of 7 RCTs, Xiao et al. [108] showed that the rate of OHSS was significantly lower in the GnRH antagonist group than the GnRH agonist group in women with PCOS (odds ratio 0.36, 95% confidence interval 0.25–0.52).

Alternatively, the risk of OHSS can be reduced by triggering final oocyte maturation with a GnRH agonist. The reduction of the risk of OHSS using a GnRH agonist trigger has been discussed above.

Another new method of tertiary prevention of early-onset OHSS using GnRH antagonists has been reported by Lainas and colleagues [109]. Antagonist administration was re-initiated at a daily dose of 0.25 mg after patients developed early OHSS, and continued daily for a week, while all embryos were cryopreserved. No progression of severe early OHSS was observed in any of the patients and none of the patients required hospitalization.

### Neonatal outcomes

Long-term outcomes after GnRH antagonist treatment do not differ from those observed with GnRH agonist regimens. Obstetrical and neonatal data on 839 pregnancies, resulting in 969 live-born infants after ganirelix treatment were compared with a historical cohort of 753 pregnancies after long GnRH agonist (buserelin) treatment, resulting in 963 live-born infants [110]. There were no differences in maternal characteristics, fertilization method, and pregnancy and delivery complications between the ganirelix and historical GnRH agonist groups. Women experienced more multiple pregnancies in the historical GnRH agonist group (31.9%) than the ganirelix group (18.7%; *P* < 0.0001), and both groups were comparable with respect to pregnancy loss after 16 weeks’ gestation. The incidence of major congenital malformations in fetuses with gestational age ≥26 weeks was 5.0% in the ganirelix cohort versus 5.4% in the historical GnRH agonist group (odds ratio, 0.94; 95% confidence interval, 0.62–1.42).

Boerrigter et al. [111] conducted a pooled analysis of all follow-up data of the phase 2 and 3 trials for the development of ganirelix. Data on 340 ongoing pregnancies and neonatal outcomes for 432 children showed that there were no differences between the GnRH antagonist and GnRH agonist regimens with respect to pregnancy loss after 16 weeks’ gestation, and the incidence and nature of complications during pregnancy and delivery did not differ between the two groups [111]. No major differences were observed in neonatal characteristics of infants in the ganirelix and agonist groups, who had an overall mean birth weight on average of 3200 g for singletons, 2300 g for twins, and 1800–1900 g for triplets. Congenital malformations were observed in 32 of 424 (7.5%) fetuses in the ganirelix group and in 10 of 181 (5.5%) in the agonist group.

## Conclusions

We reviewed the scientific literature on the use of GnRH antagonists, concentrating on the most recently available evidence. Antagonist treatment protocols are a viable alternative to agonist treatment. The multiple-dose protocol is effective in the prevention of premature LH surge. Compared with the long agonist protocol, GnRH antagonist treatment is shorter, rapidly absorbed, rapidly reversible, requires fewer injections, and appears to require a lower amount of gonadotropins, which is likely to lead to improved patient compliance and lower costs. The lower pregnancy rate reported in some early RCTs has been offset by the findings of subsequent meta-analyses, and this is probably the result of optimization of the antagonist treatment protocol. The only contraindications to the use of GnRH antagonists for the inhibition of premature LH surges in women undergoing controlled ovarian stimulation are hypersensitivity to GnRH antagonists or pregnancy [112]. GnRH antagonists have been used safely and effectively in a wide range of patients (Table 2), such as those undergoing first-line controlled ovarian stimulation [8], those with a poor prognosis [12], and patients taking oral contraceptives to regulate menstrual cycles [97]. The antagonist flexible-dosing regimen has also shown promise among women diagnosed with PCOS [114]. Certain other patient populations might particularly benefit from ganirelix protocols, such as patients who have not responded to other controlled ovarian hyperstimulation regimens (including those with GnRH agonist) [113] or, to the other extreme, patients with a high risk of developing OHSS [93,105]. There are no adverse effects associated with a GnRH antagonist protocol on assisted reproductive technology outcomes. Due to the ability to trigger final oocyte maturation with a GnRH agonist to prevent OHSS, antagonist protocols are becoming the treatment of choice for ovarian stimulation of oocyte donors [17].

**Table 2:** Suitable candidates for GnRH antagonist treatment

***Patient populations benefiting from GnRH antagonist protocols***	
• Patients undergoing first-line controlled ovarian stimulation [8,59]	
• Patients who have not responded to other controlled ovarian stimulation regimens, including those with gonadotropin-releasing hormone agonist [113]	
• Patients with a poor prognosis [12]	
• Oocyte donors [17]	
• Patients at risk for ovarian hyperstimulation syndrome [93,105]	
• Patients with polycystic ovarian syndrome [114]	
• Patients taking oral contraceptive to regulate menstrual cycles [97]	

Overall, GnRH antagonist treatment protocols are effective, easy to use, allow flexibility of treatment and, therefore, appear to offer a promising alternative to the long-established GnRH agonist regimens for prevention of a premature LH surge during ovarian stimulation for assisted reproductive techniques.

## Competing interests

ABC has received payments for speakers’ bureaus from Schering-Plough, EMD Serono, and Ferring. CB has received payments for lectures from Merck.

## Authors’ contributions

ABC and CB participated in the drafting of the manuscript and contributed to the critical discussion. Both authors gave final approval of the version to be published.
